# The International Conference on Intelligent Biology and Medicine 2019 (ICIBM 2019): computational methods and applications in medical genomics

**DOI:** 10.1186/s12920-020-0678-1

**Published:** 2020-04-03

**Authors:** Chi Zhang, Ewy Mathé, Xia Ning, Zhongming Zhao, Kai Wang, Lang Li, Yan Guo

**Affiliations:** 1 Department of Medical & Molecular Genetics, School of Medicine, Indiana University, Indianapolis, IN 46202 USA; 2 Department of Biomedical Informatics, The Ohio State University, Columbus, OH 43210 USA; 3 Center for Precision Health, School of Biomedical Informatics, The University of Texas Health Science Center at Houston, Houston, TX 77030 USA; 4 Human Genetics Center, School of Public Health, The University of Texas Health Science Center at Houston, Houston, TX 77030 USA; 5 Raymond G. Perelman Center for Cellular and Molecular Therapeutics, Children’s Hospital of Philadelphia, Philadelphia, PA 19104 USA; 6 Department of internal medicine, comprehensive cancer center, University of New Mexico, Albuquerque, NM 87131 USA

## Abstract

In this editorial, we briefly summarized the International Conference on Intelligent Biology and Medicine 2019 (ICIBM 2019) that was held on June 9–11, 2019 at Columbus, Ohio, USA. We further introduced the 19 research articles included in this supplement issue, covering four major areas, namely computational method development, genomics analysis, network-based analysis and biomarker prediction. The selected papers perform cutting edge computational research applied to a broad range of human diseases such as cancer, neural degenerative and chronic inflammatory disease. They also proposed solutions for fundamental medical genomics problems range from basic data processing and quality control to functional interpretation, biomarker and drug prediction, and database releasing.

## Introduction

The International Conference on Intelligent Biology and Medicine 2019 (ICIBM 2019) was co-hosted by the International Association for Intelligent Biology and Medicine (IAIBM) and the Department of Biomedical Informatics at The Ohio State University on June 9–11, 2019 in Columbus, OH. A total of 164 researchers attended the conference, of which 79 were faculty/staff and 84 were trainees. The conference included four keynote lectures, four eminent scholar talks, five tutorials and workshops, twelve concurrent regular scientific sessions, and one poster session. It covered a board range of topics, including but not limited to next-generation sequencing, single cell analyses, deep learning, metabolomics, genomics, and other omics research, systems biology, medical applications and translational research involving high-throughput data, computational methods and novel applications of computational tools, and others. Among 105 original manuscript submissions, 19 research articles of interests to computational method development and applications in medical genomics were selected for the ICIBM 2019 BMC Medical Genomics Supplement Issue after with careful peer reviews. The proposed computational methods and applications cover a broad range of biomedical topics and are innovative with significant biological and clinical implications. A more detailed summary of the conference arrangement, scientific programs, and achievements were published elsewhere [1]. In this editorial, we summarize the 19 selected research articles.

### Summary of selected papers

#### Computational tool development

This supplement issue includes five papers that propose computational methods to [1] predict circuit RNA and disease association, [2] predict neoantigen from multi-omics data, [3] predict cancer types via a deep learning based model, [4] infer associations in GWAS, and [5] classify SNP data from a database of miRNA target site SNPs. These computational resources are all substantially novel modeling considerations that offer new capabilities to medical genomics research.

Li et al. developed a new computational method, namely SIMCCDA (Speedup Inductive Matrix Completion for CircRNA-8 Disease Associations prediction), which is the first work that apply the recommendation system based inductive matrix completion to predict circRNA-disease associations [2]. The circRNA-disease association was modeled by a recommendation task and solved by using speedup inductive matrix completion. Three independent circRNA disease data set and their merged one were first used to compute circRNA sequence similarity and disease semantic similarity. The two similarities were further combined to predict circRNA-disease association. The method performance was validated by cross validation and application on breast, stomach and colorectal cancer data.

Li et al. developed a novel computational workflow for customized neoantigen prediction and selection [3]. The workflow takes RNA-seq, genomic sequencing and customized proteomics data as inputs and is composed by data processing, NetMHCpan based neoantigen prediction, mutant peptides filtering and selection, proteogenomics and mutant peptidome data based neoantigen filtering, and further selection of the most likely immunogenic neoantigen by their similarity with cross reactive microbial peptides. The method was constructed based on cancer cell line data but can be applied to solid cancer study. The method was implemented in a software package namely ProGeo-neo.

Mostavi et al. conducted a novel application of convolutional neural network model for cancer type prediction by using gene expression data [4]. Novel CNN designs and convolution kernels for cancer type and normal tissue type prediction were developed and compared. Application to 33 TCGA cancer types demonstrated the method can accurately predict tissue types before and after excluding tissue specific genes. A new model interpretation scheme was also developed to evaluate the importance of each gene feature in the predictive model.

Cai et al. developed a new association test, namely weighted Adaptive Fisher (wAF) to test the association of common and rare SNVs and detect dense and sparse signals in GWAS [5]. wAF is an improved version of their past Adaptive Fisher method, which achieved comparable or better statistical power when comparing with other state-of-the-art methods. Application of wAF to a schizophrenia dataset demonstrated the method can successfully detect thirteen disease associated gene, out of which nine can be support by published data.

Foroughi pour et al. developed a new computational capability to conduct binary classification based on high dimensional SNP data [6]. Binary classification based on SNP data is challenged by the small samples size, high feature space of SNP data, weak effect of SNP and possible non-linear interaction among SNPs. Based on the theory of high dimensional model representation, LABS-HDMR-CO was developed that produces classification rules with good prediction performance that can take several hundred SNPs as input, and account for their pairwise interactions. LABS-HDMR-CO is a very fast algorithm in nature, with runtime comparable to a GLM with LASSO penalty. The method evaluation suggested that high dimensional model representation can be a suitable framework to study SNP data as categorical variables.

#### Genomics analysis

Three genomics analysis are included in this supplement issue, two utilized network-assisted methods to explore new SNPs in multiple sclerosis and Cleft lip with/without cleft palate, while the other focused on somatic mutations in genetic regulatory elements in melanoma. Each of the two genomics data based study identified significant sets of biological and clinical associated genetic events.

Zhang et al. explored the landscape of somatic synonymous mutations in genetic regulatory elements in melanoma [7]. This study elucidated the role of somatic synonymous mutations in genetic aetiology of melanoma, by focusing on mutations related to five mechanisms, namely splicing regulation, transcription factor binding, miRNA binding, codon optimality, and the RNA second structure. Their analysis suggested that mutational patterns of synonymous mutations in melanoma are mostly involved in exonic splicing regulators near splicing sites or inside DNase I hypersensitivity sites or non-optimal codon. This research confirmed possible functional impact of synonymous mutations in the disease progression.

Manuel et al. conducted a network-assisted search in two multiple sclerosis GWAS data sets to identify the gene networks that were associated with the disease [8]. With applying their in-house method, dmGWAS (dense module searching of GWAS), about 7500 significant network modules were identified in at least one data set and 20 were identified from both data sets. The hub genes and functional role of each module were further studied. Direct linking between multiple sclerosis relevant biological processes, drug targets and the functions of identified genes were observed.

Yan et al. conducted a integrative network-assisted GWAS study of the Cleft lip with/without cleft palate (CL/P) [9]. By using applying their in-house methods dmGWAS (dense module searching of GWAS) and EW_dmGWAS (Edge-Weighted dmGWAS), in a combination with GWAS data, human protein-protein interaction network and differential gene expression were interactively analyzed. 87 genes were consistent identified from the sample with different ancestries by dmGWAS, out of which 27 showed nominal significance with CL/P. 253 and 245 module genes associated with CL/P from European and Asian ancestry were identified by using EW_dmGWAS. Differential expression and functional enrichment of the identified genes were further studied. Nine novel candidate genes involve in cell adhesion, plasma membrane, and regulation of apoptosis were revealed as candidate genes related to CL/P.

#### Network-based analysis

Four biological network based studies, including two differential co-expression analysis and two network based drug prediction, are included in this supplement issue. Two studies identified variations in co-expression networks through different disease stages of Alzheimer’s disease and chronic kidney disease, which can be potentially used as biomarkers of disease progression. Two novel network based drug prediction method was developed by predicting variations in signaling pathway activities in patient subgroups and applied to identify novel drug and targets in pancreatic and ovarian cancer.

Upadhyaya et al. conducted a differential gene co-expression analysis of gene expression data collected from patients’ plasma samples of Alzheimer’s disease [10]. The authors first derived co-expression networks from the data of the patients with different progression stage of Alzheimer’s disease, by using their in-house developed method. A modified joint graphical lasso model was applied to fit the authors’ assumption that the co-expression networks in consecutive disease stages are largely similar yet with critical differences. Differential co-expression analysis revealed the network clustering coefficients are stable from cognitively normal to late mild cognitive impairment stage, and further significantly decreased in Alzheimer’s disease patients, which can be potentially used as biomarkers for early screening of Alzheimer’s disease.

Yu et al. conducted a differential co-expression analysis of the RNA-seq data of chronic kidney disease (CKD) collected from patients at different disease stages [11]. Kidney tissue samples were collected from 140 patients with five different stages of CKD and 25 health donors for RNA-sequencing. Differential co-expression was conducted by using the DCGL package to identify differentially co-expressed gene pairs, modules and rewired pathways. A global attenuation of gene co-expression network was observed through the samples of different CKD stages. Specifically, strong intra-pathway correlation rewiring has been seen in the pathways including regulation of *nuclear SMAD2/3 signaling and signaling events mediated by focal adhesion kinase,* 27 including *Regulation of nuclear SMAD2/3 signaling*. The study also identified a list of vanishing hub genes and disrupted correlations within and between key signaling pathways, on the pathophysiological mechanisms of CKD progression.

Liu et al. conducted a network based study to identify possible drug targets in pancreatic ductal adenocarcinoma [12]. A novel computational method namely “spectral clustering for network-based target ranking (SCNrank)” was developed to prioritize drug targets. Specifically, SCNrank integrates three data sources namely gene expression data collected from normal and cancer tissue and cancer cell line samples, protein-protein interaction network and CRISPR-CAS9 screening data. A drug target influence score was computed from a four-step approach by computing the importance of each gene on a PPI network fitted with the gene expression data. Application of the method on pancreatic ductal adenocarcinoma identified 367 targets of FDA approved drugs, which was validated by a strong overlap between the predicted gene-drug relationship and the known drug target information.

Zhang et al. conducted an integrative network analysis to identify potential drug targets of ovarian cancer [13]. In their computational pipeline, a Markov Chain Monte Carlo method was first applied to identify up-regulated genes and potentially activated transcriptional factors in each individual patient. The ovarian cancer patients were further divided into three sub-groups based on the inferred transcriptional factor activity level. Up-stream signaling pathways of the activated transcriptional factors in each patient group were further inferred. 66 FPD approved drugs were identified targeting on the uncovered core signaling pathways, out of which 44 drugs have been reported in ovarian cancer related reports. This work developed new insights of the potential utilization of signaling diversity and heterogeneity for therapeutic targets and drug combination discovery.

#### Biomarker prediction

A substantial part of this supplement issue is composed by biomarker predictions, including seven works with utilizing [1] deep learning model to handle non-linear dependency, [2] selection and [3] prediction of transcriptional regulatory features, augmenting feature space by [4] pseudo genes and [5] network features for predicting cancer patients’ overall and disease progression free survival, and one work focused on identifying differential alternative splicing events in HIV infected T cells. Their novel modeling considerations achieved significantly increased prediction performance comparing to classic models and identified sets of new biomarkers.

Huang et al. developed a novel auto-encoder based model, namely AECOX, to identify prognostic marker genes from cohort transcriptomics data [14]. Comparing to classic models, AECOX utilized a novel auto-encoder-based formulation to derive non-linear features from the transcriptomics data that can well explain the low rank structure encoded in the data. AECOX achieved a high predictive power to the patients’ prognosis. The method was benchmarked on TCGA data and compared with existing deep learning based prognostic marker identification methods. This study demonstrated that deep learning based models, which cancer better handle the non-linear dependency between molecular and clinical features, outperform classic models in predicting patients’ prognosis by using transcriptomics data.

Liu et al. developed a novel computational pipeline to predict colon cancer prognosis by using gene expression level of transcriptional factors [15]. Univariate Cox regression model is first utilized to identify prognostic predictive transcriptional factors. A random forest based feature selection is further conducted to identify the top prognostic predictive transcriptional factors and construct an ensample model. The method was benchmarked on a collection of 4 high quality colon cancer gene expression data sets consisting 925 samples. A predictive model based on five transcriptional factors was derived, which consistently achieved a high prediction power over the four analyzed data sets analyzed.

Dong et al. developed a new computational pipeline to identify breast cancer patients’ prognosis associated transcriptional regulatory factors (TFs), by using estimated activity level of TFs [16]. Data set specific regulon was first inferred by using SCENIC and ENCODE ChIP-seq data, based on which the activity level of TFs can be further estimated. A multivariate Cox’s regression model was computed by using the estimated activity level of TFs with a step-wise variable selection approach. Application of the method on TCGA breast cancer data identified 15 transcriptional regulatory factors associated patients’ prognosis. Validations on 29 independent microarray data sets demonstrate a high reproducibility of this method.

Smerekanych et al. conducted a systematic identification of gene expression, pseudogene expression, miRNA expression, and pseudogene-gene interactions and clinical factors that were predictive to breast cancer patients’ prognosis [17]. Based on a regression model with L1 penalty, the authors identified the over expression of STXBP5, GALP and LOC387646, the up regulation of pseudogene CTSLP8 and RPS10P20 and down regulation HLA-K, the pseudogene-gene interaction between GPS2 and GPS2P1, and the microRNA miR-3923 were significantly associated with the overall survival of breast cancer. The systematic search of biomarkers from multi-omics data source offered a more precise predictive model of patients’ survival. This study demonstrated the potential usage of pseudogene as cancer prognosis markers.

Adnan et al. established a comparative evaluation of network features to predict the metastasis of breast cancer on data collected in 12 cohorts [18]. The authors compared several types of network features, including gene expression and gene variations weighted by their neighbors obtained from protein-protein interaction network, and gene co-expression networks to predict breast cancer metastasis. The underlying hypothesis of their data augmentation approach is that a gene expression feature weighted by other neighbor genes over a biological network may quantify the activity level of a local part in a biological process. A univariate analysis was first conducted to identify the features that were predictive of breast cancer metastasis. An ensemble classification was further developed using the selected network features. A consistent improvement of prediction performance using network based features was observed.

Zhang et al. conducted a pan-cancer study to demonstrate the clinical implication of class-3 semaphorins in the treatment of cancer [19]. By computing the differential expression of class-3 semaphorins and their association with patients’ overall survival in 31 TCGA cancer types, the authors identified SEMA3A, SEMA3C and SEMA3F are consistently up-regulated and the expression of SEMA3 family members were frequently associated with patients’ prognosis in multiple cancer types. In addition, a significant association between SEMA3 family and immune cell infiltration has been observed. The analysis suggested SEMA3C and SEMA3F may potentially contribute to the resistance of certain drugs.

Byun et al. conducted a study of the differential alternative splicing in HIV infected T cells [20]. Application of rMATs on publicly available RNA-seq data of HIV infected and normal control T cell, the authors identified 427 candidate genes with differentially expressed alternative splicing exons in infected T-cells, including 20 genes related to cell surface, 35 to kinases, and 121 to immune-related genes. Further pathway analysis and experimental validations confirmed the biological implications of the alternative splicing genes. To the best of our knowledge, this study is first work to systematically explore the alternative splicing events in HIV infected T cells, which provides novel insights of possible clinical implications of targeting the differential alternative splicing events in treating the disease.

## Discussion

ICIBM conferences provide a friendly forum for researchers to present and publish cutting-edge biomedical studies. Stimulated by state-of-the-art biotechnologies, therapeutic strategies, biological hypotheses and interdisciplinary capabilities, computational scientists developed models to link biomedical data with biological hypothesis from different perspectives. Here, the 16 selected works illustrating innovative computational ideas or applications of new computational methods on important biomedical questions. Even though the high complexity of some models may limit their application in a broader domain and some observations still need further experimental validations, we anticipate some of these biomedical studies and computationally derived results can contribute in real clinical applications, as is discussed in this supplemental issue.
